# Is heat wave a predictor of diarrhoea in Dhaka, Bangladesh? A time-series analysis in a South Asian tropical monsoon climate

**DOI:** 10.1371/journal.pgph.0003629

**Published:** 2024-09-03

**Authors:** Farhana Haque, Fiona C. Lampe, Shakoor Hajat, Katerina Stavrianaki, S. M. Tafsir Hasan, A. S. G. Faruque, Tahmeed Ahmed, Shamim Jubayer, Ilan Kelman

**Affiliations:** 1 Institute for Global Health (IGH), University College London (UCL), London, United Kingdom; 2 UK Public Health Rapid Support Team (UK-PHRST), Department of Infectious Disease Epidemiology, London School of Hygiene and Tropical Medicine (LSHTM), London, United Kingdom; 3 Department of Public Health, Environments and Society, London School of Hygiene and Tropical Medicine (LSHTM), London, United Kingdom; 4 Department of Statistical Science, Department of Risk and Disaster Reduction, University College London (UCL), London, United Kingdom; 5 Nutrition and Clinical Services Division, icddr,b, Dhaka, Bangladesh; 6 National Heart Foundation Hospital and Research Institute (NHF&RI), Dhaka, Bangladesh; 7 Department of Risk and Disaster Reduction, Institute for Global Health (IGH), University College London (UCL), London, United Kingdom; 8 University of Agder, Kristiansand, Norway; Keele University, UNITED KINGDOM OF GREAT BRITAIN AND NORTHERN IRELAND

## Abstract

While numerous studies have assessed the association between temperature and diarrhoea in various locations, evidence of relationship between heat wave and diarrhoea is scarce. We defined elevated daily mean and maximum temperature over the 95^th^ and 99^th^ percentiles lasting for at least one day between March to October 1981–2010 as TAV95 and TAV99 and D95 and D99 heat wave, respectively. We investigated the association between heat wave and daily counts of hospitalisations for all-cause diarrhoea in Dhaka, Bangladesh using time series regression analysis employing constrained distributed lag-linear models. Effects were assessed for all ages and children aged under 5 years of age. Diarrhoea hospitalisation increased by 6.7% (95% CI: 4.6%– 8.9%), 8.3% (3.7–13.1), 7.0 (4.8–9.3) and 7.4 (3.1–11.9) in all ages on a TAV95, TAV99, D95 and D99 heat wave day, respectively. These effects were more pronounced for under-5 children with an increase of 13.9% (95% CI: 8.3–19.9), 24.2% (11.3–38.7), 17.0 (11.0–23.5) and 19.5 (7.7–32.6) in diarrhoea hospitalisations on a TAV95, TAV99, D95 and D99 heat wave day, respectively. At lags of 3 days, we noticed a negative association indicating a ‘harvesting’ effect. Our findings suggest that heat wave was a significant risk factor for diarrhoea hospitalisation in Dhaka. Further research is needed to elucidate the causal pathways and identify the preventive measures necessary to mitigate the impacts of heat waves on diarrhoea. Given that no heat wave definitions exist for Dhaka, these results may help to define heat waves for Dhaka and trigger public health interventions including heat alerts to prevent heat-related morbidity in Dhaka, Bangladesh.

## Introduction

It is now evident that anthropogenic climate change is increasing the intensity and frequency as well as duration of heat waves in addition to raising the average ambient temperature across the globe [1, 2]. The observed increasing trend of heat waves and warm spells due to global climate change are projected to continue in the future [3, 4]. Heat waves can exert serious and potentially life-threatening impacts on human health including heat stroke, heat exhaustion, heat syncope, and heat cramps [5].

Health effects of heat wave tend to be governed by a variety of complex, interacting biological, medical, environmental, social and geographical factors including locations, individual susceptibility, prevalence of certain diseases, healthcare infrastructure and health system status [2, 5–7]. In addition, the mechanisms by which extreme temperatures influence disease causation may vary widely according to different morbidities. For example, heat extremes in countries with less than optimum water and sanitation infrastructure may significantly increase the risk of waterborne diseases including diarrhoea by increasing exposure to contaminated drinking water needed to replace the volume lost through excessive sweat in addition to increasing host susceptibility to infection [5].

Although there are several reports of increased mortality, limited information exists on the impact of heat waves on morbidity across the globe and particularly in the South Asian context [8, 9]. The perceived risk of health hazards from heat waves or warm spells is low in the developing countries of the tropical and sub-tropical regions in South Asia where comfortable warm temperature is the norm [9]. Although temperature-related deaths and diseases may be largely preventable and heat warning systems (HWSs) as well as heat early warning systems (HEWS) are existent in many high-income cities globally, such warning systems rarely exists in the South Asian setting [10]. One important gap that hampered the development of a warning system in South Asian countries is the lack of consensus about the definition of heat waves. Furthermore, there is dearth of knowledge regarding the nature of heat-health risk, climate hazard, societal exposure and population vulnerability [9].

Diarrhoea occurs when a person passes more than three loose, watery stools within 24 hours. Although the exact prevalence and incidence of diarrhoea are not available, diarrhoea is endemic in Bangladesh [11]. With improvements in socio-economic conditions, water and sanitation infrastructure and diarrhoea care, Bangladesh has achieved significant success in reducing diarrhoea mortality in the recent years [12, 13]. However, diarrhoea outbreaks and hyper-endemicity continue to plague the nation [11, 14]. On the other hand, Bangladesh, a South Asian country with a tropical monsoon climate, is highly vulnerable to the adverse impacts of climate change [15] and heat waves in the future [9]. With more than an estimated 123.47 million diarrhoeal disease episodes in all age groups in Bangladesh annually in 2016 [11], the potential impact of heat wave on the incidence of diarrhoeal disease in the future could be concerning for Bangladesh [16–19]. Given that the capital city of Dhaka is struggling to ensure water quality and facing a number of challenges to ensure the quality of urban life and sustainable urban growth including rising surface temperature in the context of urbanisation and global climate change, insufficient infrastructure, inadequate sanitation and poor hygiene brought about by poverty [20, 21], the impacts of heat waves on diarrhoea are likely to be considerably high in Dhaka [17].

This paper aims to evaluate the influence of heat waves on hospitalisations due to diarrhoea in Dhaka. Although it is acknowledged that correlations uncovered do not necessarily imply direct causation, such indicators support understanding of the effects of heat waves on diarrhoeal disease morbidity thereby aiding further research to elicit linkages between climate change and gastrointestinal health. Given that many of the South Asian cities including Dhaka do not currently have a clearly agreed heat wave definition, this paper additionally aimed to identify pragmatic definitions of heat waves for Dhaka, which is a necessary first step to inform the development of a HWS for Dhaka.

## Data and methodology

### Diarrhoea data

Daily diarrhoea hospitalisation data between 1 January 1981 to 31 December 2010 were collected from the Dhaka Hospital of the International Centre for Diarrhoeal Diseases Research, Bangladesh (icddr,b) on 7 October 2020. The Dhaka Hospital is a well-known hospital and the largest health facility providing specialist care to diarrhoea patients [22, 23]. The hospital served an urban population of approximately 3.5 million in 1981, 6.6 million in 1990 and 14.6 million in 2010 and provided free treatment to more than 140,000 patients with diarrhoea in 2010 [24]. Given that reliable records of the total number of patients admitted with diarrhoea per day or their disease onset dates were not available from Dhaka Hospital or any other hospital for the study period (1981–2010), information from the robust Diarrhoeal Disease Surveillance System (DDSS) was obtained instead to estimate the total number of patients hospitalised with diarrhoea per day. We did not access any information that could identify individual participants during or after data collection. The DDSS platform recorded the information of all-cause diarrhoea patients who were enrolled into the surveillance system [23]. It is likely that predominantly infectious gastroenteritis (IG) cases were included in this study. However, a limited number of people who had chronic or persistent diarrhoea at their first presentation and people with inflammatory bowel disease (IBD) who presented with similar symptoms were also likely included. Since ambient temperature including heat wave affect both IG and IBD [8], and because it was logistically impossible to test all stool samples for all possible pathogens, a syndromic approach was regarded appropriate for this study (S1 File).

### Meteorological data

We collected data on daily climate parameters including the ambient, maximum, minimum temperature (°C), cumulative rainfall (mm), and relative humidity (%) for Dhaka City from the Bangladesh Meteorological Department (BMD) from 1981–2010. The BMD recorded 3-hourly data from three validated weather stations for Dhaka (https://bmd.gov.bd/external-link/https://dataportal.bmd.gov.bd/).

### Defining heat wave for Dhaka

While many organisations, countries and research studies have proposed various definitions, a globally acceptable definition of heat wave remains elusive [9, 10, 25–27]. Ideally, thresholds for operational heat wave definitions should be determined according to the local climate and by incorporating the vulnerability of the local population. However, many proposed heat wave indicators do not incorporate population vulnerability [10]. The World Meteorological Organization classified any period of 6 days with maximum temperature >5°C above the daily average maximum temperature as a heat wave [10, 25, 26]. One report from the BMD classified the heat wave for Bangladesh as follows: mild heat wave (when the maximum temperature lies between 36–38°C), moderate heat wave (when maximum temperature lies between 38–40°C), and severe heat wave (when maximum temperature is greater than 40°C) [28]. However, these definitions are based on meteorological criteria and are not tailored for issues of public health. In contrast, a study using the nationwide daily death counts from the Sample Vital Registration System (SVRS) of the Bangladesh Bureau of Statistics (BBS) proposed a heat wave definition for Bangladesh as the elevated minimum and maximum daily temperature above the 95th percentile [9]. Given that the health impact may increase nonlinearly with persistence of heat waves, duration of two or more days have been proposed in the definition of heat waves in many countries across the globe [9, 10]. Heat wave in France is declared when daily maximum and minimum temperatures remain elevated for 3 days but the thresholds are generated for each sub-region according to local mortality data [10]. On the other hand, a study from India considered heat waves to be three or more consecutive days of temperatures above the 85th percentile of the hottest month for each specific location [29].

In the absence of an acceptable and agreed definition of heat wave for Dhaka, Bangladesh, we calculated 16 simple indices of heat wave for Dhaka based on percentiles and duration only. Since both day and night time temperature are known to affect thermal stress, some of the proposed indicators considered both day (maximum) and night time (minimum) temperature. Since our preliminary analysis showed that most of the heat wave events during the study period lasted for ≤3 days, we proposed the indicators lasting for 1–3 days. These are summarised in Table 1. Analyses of heat extremes were restricted to the warm seasons (pre-monsoon summers and rainy monsoons–March–October) to avoid confounding by cold temperature [9].

**Table 1:** Definitions of the 16 proposed heat wave indicators tested. Max and min represent the daily maximum and minimum temperature, respectively. All indices calculated from March to October during 1981–2010.

Index name	Conditions	Minimum duration (day)
**TAV95**	Daily mean temperature > 95^th^ percentile	1
**TAV99**	Daily mean temperature > 99^th^ percentile	1
**D95**	Daily max temperature > 95^th^ percentile	1
**D99**	Daily max temperature > 99^th^ percentile	1
**MIN95**	Daily min temperature >95^th^ percentile	1
**D&N**	Daily max and min temperature >95^th^ percentile	1
**TAV952**	Daily mean temperature > 95^th^ percentile	2
**TAV953**	Daily mean temperature > 95^th^ percentile	3
**TAV992**	Daily mean temperature > 99^th^ percentile	2
**TAV993**	Daily mean temperature > 99^th^ percentile	3
**D952**	Daily max temperature > 95^th^ percentile	2
**D953**	Daily max temperature > 95^th^ percentile	3
**D992**	Daily max temperature > 99^th^ percentile	2
**D993**	Daily max temperature > 99^th^ percentile	3
**MIN952**	Daily min temperature >95^th^ percentile	2
**D&N2**	Daily max and min temperature >95^th^ percentile	2

### Exploratory analysis

Any missing data on the climate or health parameters were replaced by the by the respective month’s average value for the parameter. Using established methods, each data series were checked for stationarity, autocorrelation, long-term trends, seasonality, possible outliers, normality, homoscedasticity and volatility [30–32].

### Regression modelling

Negative binomial time series regression models were employed to compute the incidence rate ratio (IRR) estimates for the immediate and lagged effects of heat waves (explanatory variables) on daily diarrhoea hospitalisations (response variable). We checked for overdispersion in the data using methods described in Hardin and Hilbe (2018) [33]. The negative binomial distribution was chosen to allow us to appropriately model the extra-variations (overdispersion) in the response variable [32–34]. The Wald-type 95% confidence intervals for the incidence rate ratios and associated *P*-values based on a reference distribution were also computed. The simple heat wave indicators defined above were used as predictor variables, and the regression models were used to determine the percentage increase or decrease in diarrhoea morbidity associated with each indicator. The risk estimates were adjusted for day of the week effects, long-term time trend and seasonality (using natural cubic splines) and autocorrelation. Since access to healthcare and diarrhoea hospitalisation varied between weekdays and weekends, we adjusted the model for the day of the week effects [32, 35–40]. A categorical variable for the day of the week with 7 categories, treating public holidays as Fridays (which represented the weekend holiday in Bangladesh) was incorporated into the model to account for the artificial drop in hospital visits during the weekend as opposed to weekdays [37]. For this study, the long-term trends and seasonality was mainly controlled by fitting a spline function of time as part of the regression model. The spline function of time represented the number of different polynomial curves joined smoothly end-to-end to cover the study duration. To fit a spline function, a set of basis variables that were functions of the main time variable was generated for inclusion into the regression model. Before generating the spline basis, the number of knots that governed flexibility of the curve by creating a number of end-to-end cubic curves need to be decided. Too few knots risked failure to detect the main long-term patterns whereas too many knots could make the function unstable, which could compete with the explanatory variables and extend the confidence intervals of relative risk estimates. Given that the optimum degree of freedom per year to account for the long-term trend and seasonality was unknown, the analysis was repeated with 3–7 degrees of freedom per year. The model with the lowest BIC value was the preferred model.

Past studies have shown significant effects of heavy rainfall and inconsistent effects of relative humidity on diarrhoea [36, 41–49]. Heavy rainfall increases the occurrence of diarrhoeal disease by increasing contamination of drinking water. Heavy rainfall increases host susceptibility to infection by causing malnutrition through crop/livestock destruction and reduced agricultural yield [44]. As a result, the heat wave models were adjusted for heavy rainfall and humidity was not included in the models. Heavy rainfall (defined as the rainfall above the 95th percentile for the study period) was included as a categorical variable. Past studies have also highlighted potential lag effects of heavy rainfall on diarrhoea. Since individual and distributed lagged models allowed investigation of potential harvesting effects, correlation analysis was performed with relevant lag values of temperature extremes. As statistically significant relationship between heat wave at lags of 0 and 3 days and diarrhoea were found, lag effects were considered in the final model. Lagged effects of heavy rainfall (0–8 days) were also included into the model. Ultimately, constraints were included into the distributed lag linear model (DLLM) to investigate the effects of heat waves on diarrhoea after adjusting for the potential confounding effects of heavy rainfall, long-term trend, seasonality, day-of-the-week effect and autocorrelation. The constrained DLLM allowed us to overcome the problem of collinearity in the model and led to precise estimates of confidence intervals [50]. Therefore, the total number of hospitalisations due to diarrhoea per day was considered as the response variable and immediate and lagged effects of heat wave and heavy rainfall, day of the week and natural cubic spline of time as the explanatory variables for the regression analysis.

The final model took the following form:

*Y*_*t*_ *~ Negative Binomial (μ*_*t*_, *θ)*

$$\log[E(Y_{t})]=\beta_{0}+\sum \beta_{1p}ET_{t-1p}+\sum \beta_{2q}HeavyRain_{t-2q}+\sum NS(time_{t},7DF)+\sum \beta_{3}Dow$$ (1)

where Y_t_ denoted daily all-cause diarrhoea count, ET_t_ and HeavyRain_t_ denoted heat extreme/wave and heavy rainfall indicator at time t. To control for long-term trends and seasonality, a natural cubic spline (NS) of time with 7 degrees of freedom per year was incorporated into the model. Dow_t_ was the categorical day of the week with a reference day of Friday.

The relative risk of hospitalisation for all-cause diarrhoea during a heat wave day was calculated from Eq (1) as incidence rate ratio (IRR) and the associated percentage increase in hospitalisation during heat wave days were derived from the model parameters through Eq (2)

%change=100(IRR−1) (2)

To elicit the modulating effects of age on the relationship between heat wave and diarrhoea, separate stratum-specific analyses were conducted for diarrhoea hospitalisations for all age groups and children under 5 years of age using the Eqs (1) and (2). Multiple sensitivity analyses by changing the amount of control for seasonality and long-term trend, including relative humidity as a linear term and heavy rainfall as a categorical variable without any lagged effects were carried out to check if the main findings were robust to changes in key assumptions. In addition, the analyses were rerun using the total number of diarrhoea patients enrolled into the icddr,b DDSS as the outcome instead of the total estimated diarrhoea hospitalisations per day. We used Stata/SE 16.0 (StataCorp LLC) for data analyses in this study.

### Ethics statement

This study was granted approval by the Research Review Committee (RRC) and Ethical Review Committee (ERC) of icddr,b (PR-19097). The study used secondary data and did not involve primary data collection from human participants. The study was also approved by the UCL Research Ethics Committee (UCL REC).

## Results

**Fig 1:**
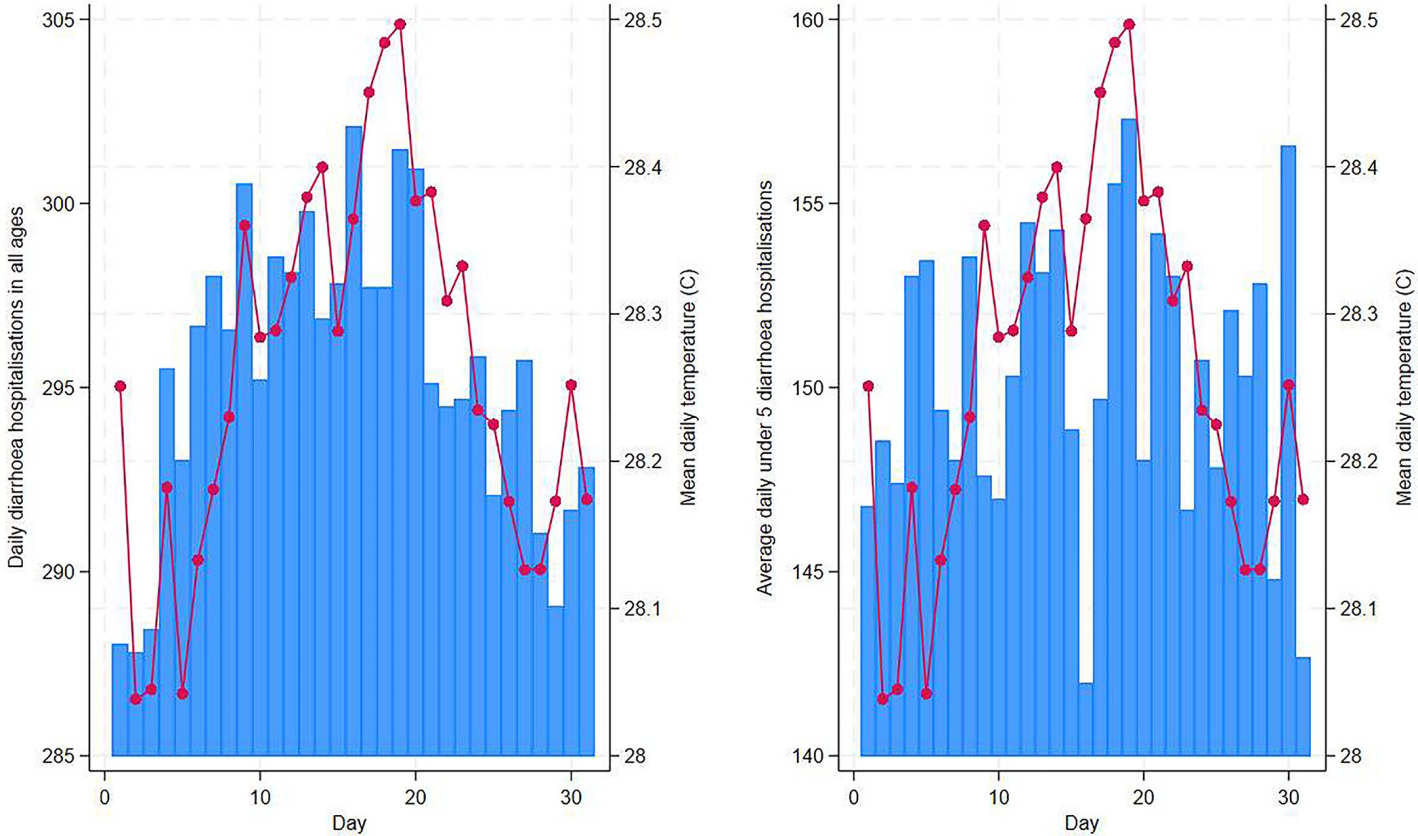
Daily distribution of diarrhoea hospitalisation in all ages (left) and in children under 5 years of age (right) and ambient temperature averaged across years 1981–2010 in Dhaka, Bangladesh.

**Fig 2:**
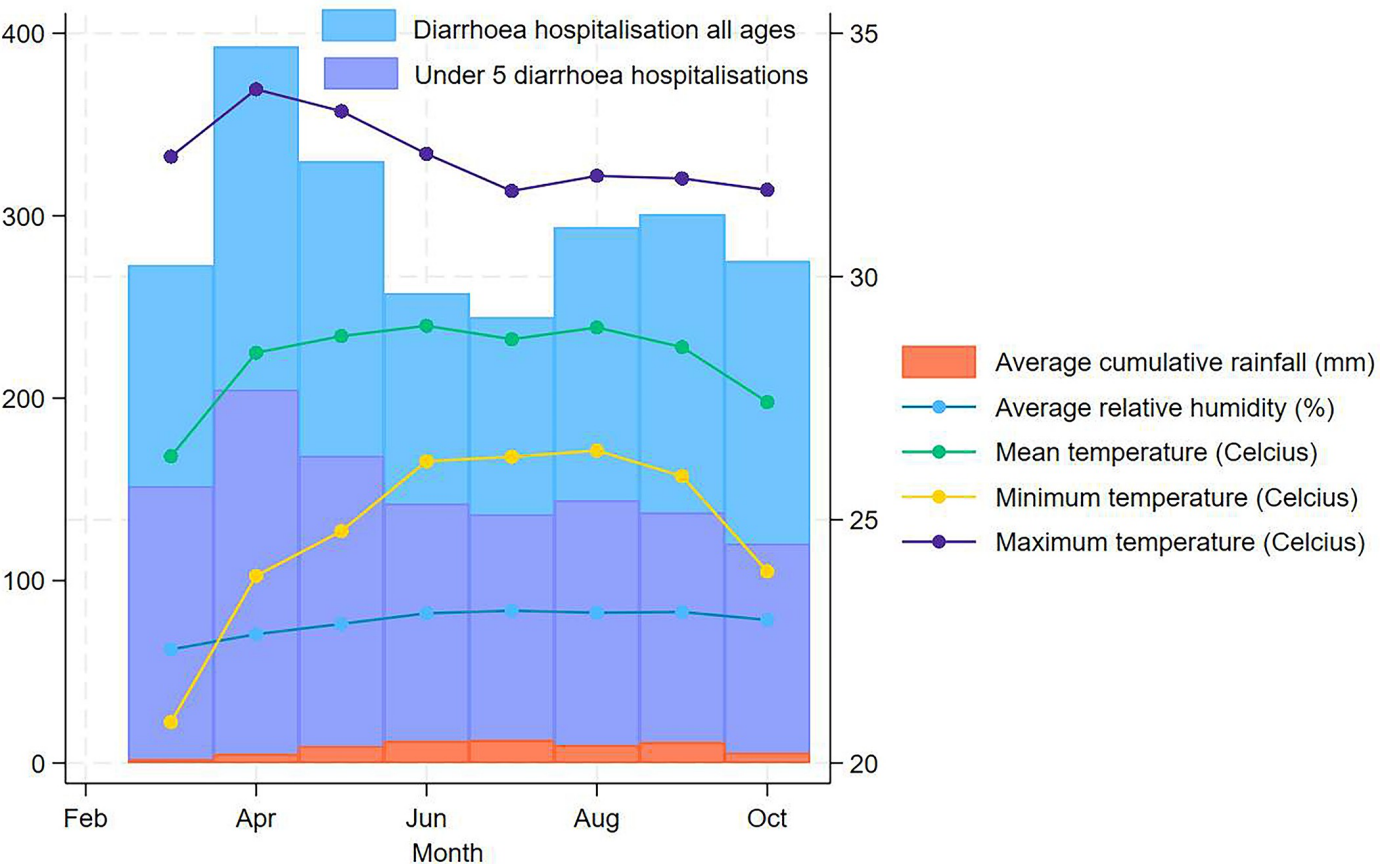
Monthly distribution of daily diarrhoea hospitalisation in all ages and ≤5 children and meteorological factors averaged across years 1981–2010.

Between March to October 1981–2010, a total of 61,054 diarrhoea cases were enrolled into the DDSS platform and an estimated total of 2,171,500 patients of all ages and 1,103,325 children <5 years of age with all-cause diarrhoea sought hospital care from the icddr,b Dhaka Hospital. Fig 1 shows the daily and monthly distributions (March through October) of diarrhoea hospitalisation and mean temperature (°C) in Dhaka averaged across years 1981–2010. The average seasonal cycles of diarrhoea hospitalisation, temperature, cumulative rainfall and relative humidity in Bangladesh are shown in Fig 2. Diarrhoea hospitalisation for under 5 children peaked in April whereas diarrhoea hospitalisation for all ages revealed a large peak in April and a smaller peak in September (Fig 2). Mean temperature remained high from April to June peaking during June before lowering down in October. Maximum temperature reached an annual maximum in April (close to 35⁰C) and decreased markedly during the rainy monsoon (July through to October) when the relative humidity was also high. However, night time temperatures (daily minimum) did not show similar pattern as day time temperatures (daily maximum). Relative humidity reached an annual maximum at approximately 90% during July and decreased towards the end of the rainy season in October. Findings from the exploratory analysis are provided in the S2 File.

**Fig 3:**
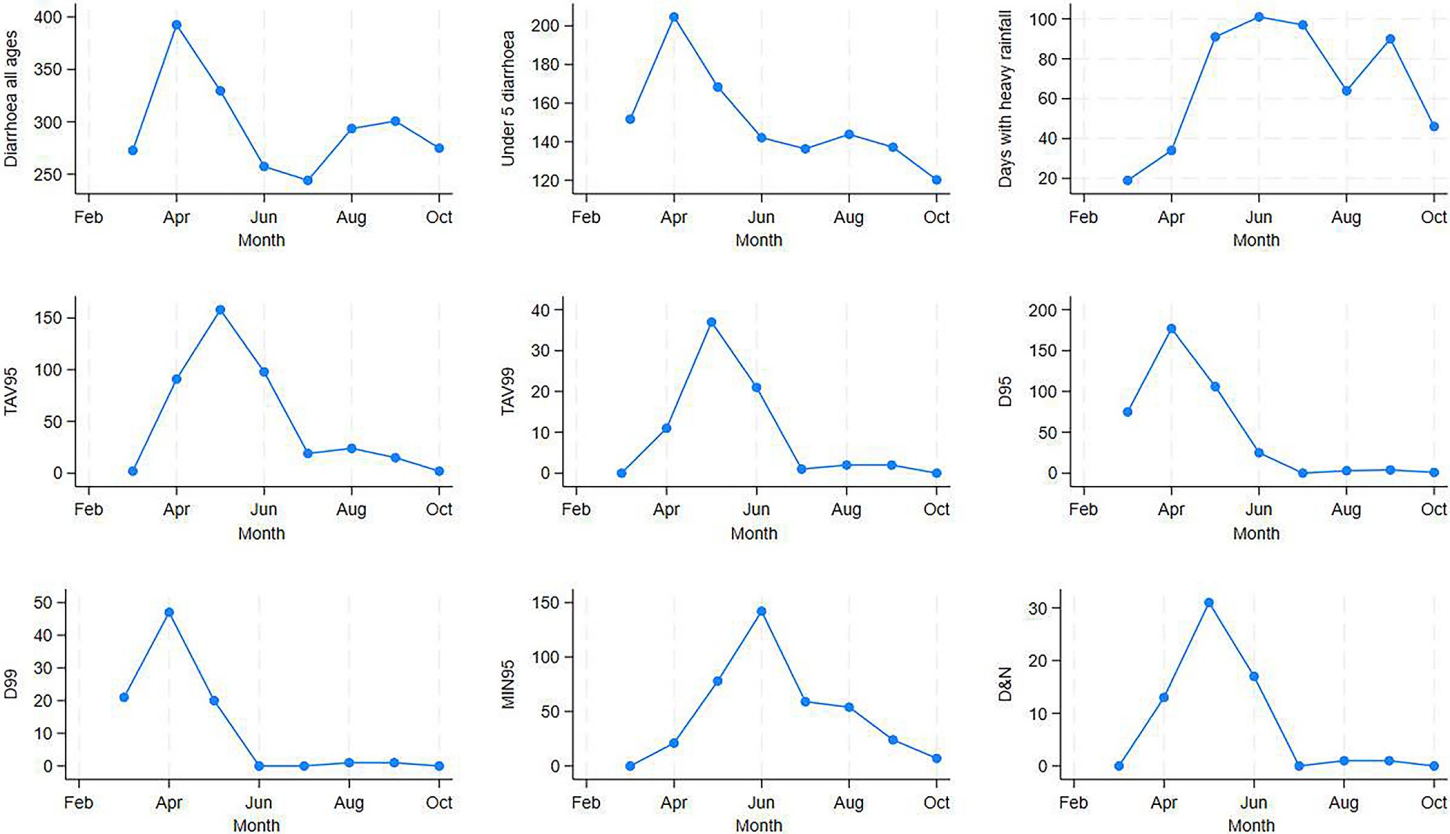
Monthly distribution of diarrhoea hospitalisations in all ages and <5 children in Dhaka Hospital, heavy rainfall and heat wave days in Dhaka, Bangladesh, March–October 1981–2010.

Fig 3 shows the temporal distribution of the heat wave day indicators by months. Most heat wave days were concentrated during the summer months. TAV95 and TAV99 heat wave categories peaked in May. D95 and D99 heat waves peaked during April. The MIN95 heat wave days were more widely distributed between April through October with the highest number found in June. The combined minimum and maximum temperature category (D&N) heat wave days were concentrated during April through June with the highest number in May.

Table 2 displays the temporal distribution of the heat wave day indicators by decades. While the TAV95, TAV 99, MIN95 and D&N heat waves appeared to be increasing, the D95 and D99 heat wave days showed a decreasing trend across the decades. Table 3 shows the persistence of the heat wave day indicators lasting for 1–13 days. In all categories, most of the heat waves lasted for one day only with very few events lasting for more than four days. Two episodes of TAV95 heat wave and one episode of D95 heat wave lasting for a maximum of 13 consecutive days were identified during the study period.

**Table 2:** Distribution of the 16 proposed heat wave indicators tested by decades. All indices calculated from March to October during 1981–2010.

Index	Number of heat-wave events			
	1981–1990	1991–2000	2001–2010	1981–2010
**TAV95**	112	130	167	409
**TAV99**	20	26	28	74
**D95**	141	144	106	391
**D99**	45	34	11	90
**MIN95**	100	127	158	385
**D&N**	17	18	28	63
**TAV952**	24	29	31	84
**TAV953**	14	17	18	49
**TAV992**	6	5	6	17
**TAV993**	0	3	4	7
**D952**	28	30	23	81
**D953**	18	20	14	52
**D992**	10	6	1	17
**D993**	5	3	1	9
**MIN952**	17	23	31	71
**D&N2**	4	3	5	12

**Table 3:** Duration of persistence of heat wave days in Dhaka, Bangladesh, 1981–2010.

Duration of persistence of heat wave (Days)	Heat wave category					
	TAV95	TAV99	D95	D99	MIN95	D&N
**1**	92	30	68	35	145	28
**2**	35	10	29	8	36	5
**3**	19	4	17	1	12	4
**4**	9	3	8	5	10	2
**5**	7	-	10	2	5	1
**6**	5	-	7	1	4	-
**7**	1	-	3	-	3	-
**8**	2	-	3	-	-	-
**9**	3	-	2	-	-	-
**10**	-	-	-	-	1	-
**11**	1	-	-	-	-	-
**12**	-	-	1	-	1	-
**13**	2	-	1	-	-	-

Table 4 displays the percentage increase in diarrhoea hospitalisation during heat wave events. We found significant increase in diarrhoea hospitalisation in all ages for only 5 out of the 16 proposed heat wave indicators. For <5 children significant results were obtained for 6 out of the 16 proposed indices. Compared to a non-heat wave day, all-cause diarrhoea hospitalisation increased by 7% and 8% in all ages and by 14% and 24% in children under 5 years on a TAV95 and TAV99 heat wave day, respectively. Increases in diarrhoea hospitalisations were strongest when defining heat waves using 99th percentile of daily maximum temperature.

**Table 4:** Percentage increase in diarrhoea hospitalisation in all ages and <5 children during heat wave days compared to non-heat wave days in Dhaka, 1981–2010.

	All ages		<5 Children	
Indicator	Percentage increase in diarrhoea hospitalisations on heat wave days (95% CI)	*P*-value	Percentage increase in diarrhoea hospitalisations on heat wave days (95% CI)	*P*-value
**TAV95**	**6.7 (4.6–8.9)**	<0.001	**13.9 (8.3–19.9)**	<0.001
**TAV99**	**8.3 (3.7–13.1)**	<0.001	**24.2 (11.3–38.7)**	<0.001
**D95**	**7.0 (4.8–9.3)**	<0.001	**17.0 (11.0–23.5)**	<0.001
**D99**	**7.4 (3.1–11.9)**	0.001	**19.5 (7.7–32.6)**	0.001
**MIN95**	0.05 (-0.2–2.1)	0.964	4.4 (-0.8–9.9)	0.098
**D&N**	4.0 (-0.8–9.1)	0.107	**14.0 (0.9–28.7)**	0.035
**TAV952**	**4.6 (0.4–9.0)**	0.031	**21.0 (3.2–41.9)**	0.019
**TAV953**	-1.3 (-9.3–7.5)	0.770	17.0 (-5.7–45.2)	0.153
**TAV992**	1.9 (-0.50–9.2)	0.599	29.4 (-3.9–74.3)	0.089
**TAV993**	5.2 (-3.8–15.1)	0.269	16.7 (-27.0–86.5)	0.519
**D952**	1.9 (-5.0–9.2)	0.599	13.4 (-4.91–35.1)	0.162
**D953**	5.2 (-3.8–15.1)	0.269	22.3 (-2.7–53.6)	0.084
**D992**	11.5 (-2.3–27.2)	0.755	22.77 (-12.1–71.3	0.230
**D993**	-34.1 (-5.2–36.5)	0.776	-11.8 (-65.4–25.2)	0.793
**MIN952**	-5.8 (-11.6–0.2)	0.058	-2.3 (-16.5–14.4)	0.755
**D&N2**	-4.8 (-19.4–12.5)	0.562	19.4 (-21.6–81.9)	0.409

*Bold values indicate significant results

Although lower than the same day effect, heat waves persisting for two days (TAV952) was significantly associated with diarrhoea among all ages. This effect was four times stronger in <5 children compared to all ages (4.6% Vs 21%). Significant effects were also observed for maximum temperature categories (D95 and D99). For all ages, neither the minimum temperature nor the days when both minimum and maximum temperature exceeded the 95th percentile (D&N) were found to be significantly associated with diarrhoea hospitalisation. However, significant effects of D&N were observed among <5 children. No significant effects of heat wave that lasted for three or more days were observed in these models.

Lagged effects of heat wave days were evaluated for 0–14 days initially in individual lag distributed models and later using constrained distributed lag linear models. Diarrhoea hospitalisation decreased by 3.5% (95% CI: 1.5%– 5.4%) three days following a TAV95 heat wave day. Significant negative effects of heavy rainfall were observed at lags 0–1 whereas significant positive effects of heavy rainfall were observed at the lags of 2–8 days. Compared to the holiday of week (Friday), diarrhoea hospitalisations were significantly higher in all weekdays with the highest effect observed on Sunday, when diarrhoea hospital increased by 10.3% (Table 5). Similarly, diarrhoea hospitalisation decreased by 4.9% (95% CI: 0.7%– 9.0%) three days following a TAV99 heat wave day.

**Table 5:** Adjusted associations among TAV95 heat wave (defined as the days with elevated mean temperature above the 95^th^ percentile) and diarrhoea hospitalisations in Dhaka, March to October 1981 to 2010^a^.

Variable	IRR	95% CI	P-value
**TAV95 Heat wave (Daily mean temperature >95**^**th**^ **percentile)**			
**Lag 0**	1.0672	1.0460–1.0889	<0.001
**Lag 1**	1.0011	0.9765–1.0263	0.930
**Lag 2**	0.9783	0.9542–1.0030	0.084
**Lag 3**	0.9650	0.9457–0.9847	0.001
**Heavy rainfall (>95**^**th**^ **percentile)**			
**Lag 0**	0.9098	0.8945–0.9253	<0.001
**Lag 1**	0.9383	0.9221–0.9548	<0.001
**Lag 2**	1.0201	1.0027–1.0378	0.023
**Lag 3**	1.0553	1.0373–1.0736	<0.001
**Lag 4**	1.0544	1.0365–1.0727	<0.001
**Lag 5**	1.0447	1.0270–1.0627	<0.001
**Lag 6**	1.0243	1.0124–1.0478	0.001
**Lag 7**	1.0268	1.0076–1.0429	0.005
**Lag 8**	1.0222	1.0046–1.0394	0.013
**Day of the week**			
**Friday**	Referent		
**Saturday**	1.0759	1.0580–1.0941	<0.001
**Sunday**	1.1033	1.0849–1.1221	<0.001
**Monday**	1.0668	1.0491–1.0848	<0.001
**Tuesday**	1.0368	1.0197–1.0543	<0.001
**Wednesday**	1.0558	1.0383–1.0735	<0.001
**Thursday**	1.0646	1.0470–1.0824	<0.001

^a^ Constrained distributed lag linear model developed using Eq 1 after controlling for long term trend and seasonality, autocorrelation, and lagged effects of heavy rainfall (0–8).

AIC = 7580.0; BIC = 7596.4; Dispersion statistic = 0.9577; Mean deviance residual = -0.0573

**Fig 4:**
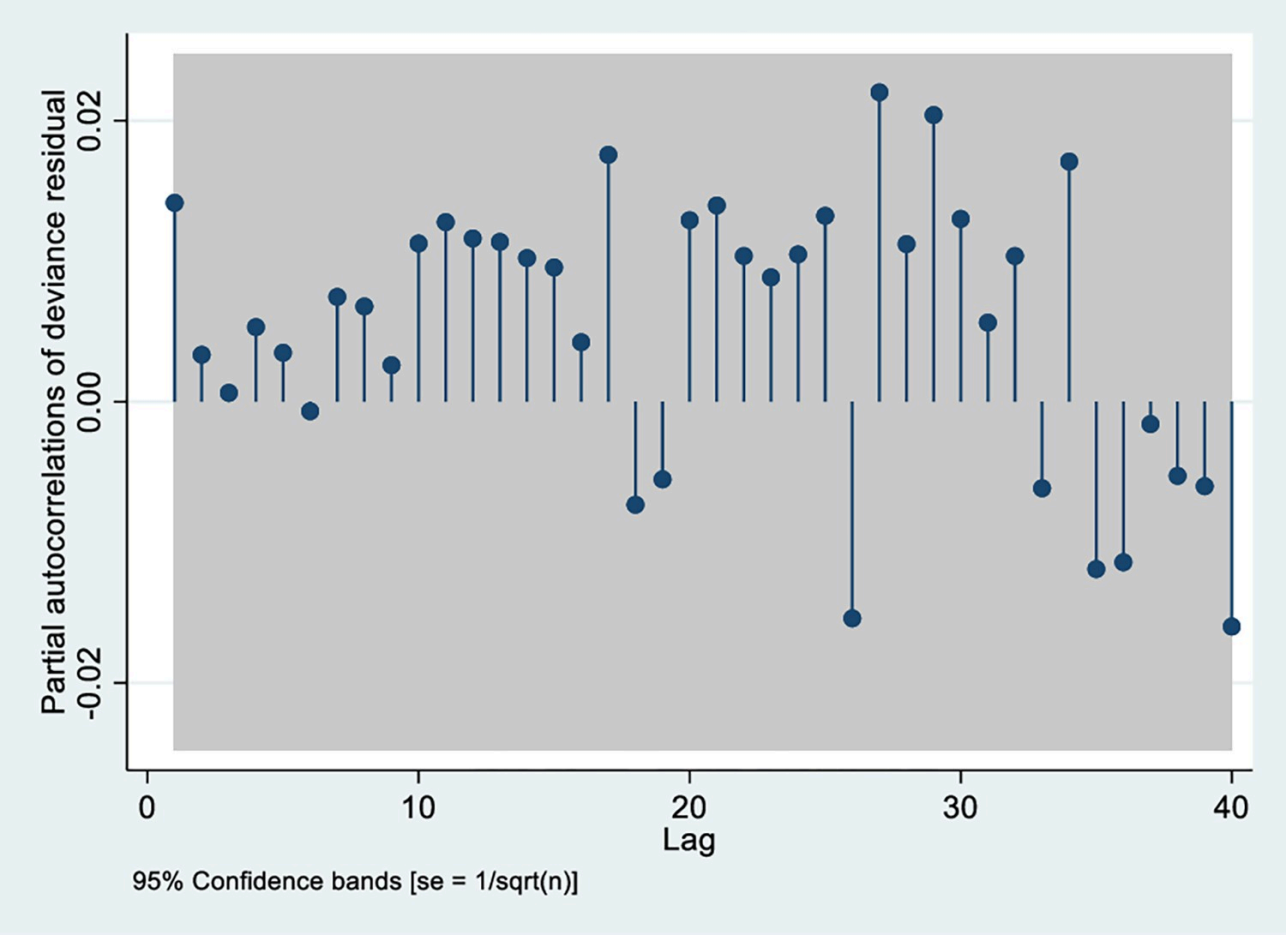
Partial autocorrelation function plot of deviance residuals of the final regression model adjusted for autocorrelation where heat wave was defined by the exceedance of 95^th^ percentile of the mean temperature.

Each model was evaluated to check model fit in addition to evaluating the residual analysis, dispersion statistic and BIC values (S3 File). Fig 4 displays the partial autocorrelation plot of deviance residuals from the final regression model depicting the relationship between TAV95 heat wave day and diarrhoea hospitalisation showing minimal residual autocorrelations. The sensitivity analysis conducted by changing the control for long-term trend and seasonality, modifying model parameters and by using total number of patients enrolled into the surveillance system instead of estimated counts of daily diarrhoea hospitalization as the primary outcome changed the results very little (S3 File).

## Discussion

This study found a statistically significant relationship between heat waves and diarrhoea hospitalisations in Dhaka, Bangladesh. This is one of the few studies to investigate the effects of heat waves on diarrhoeal disease morbidity and therefore provides essential information for analysing the potential impact of climate change on diarrhoea [8, 19, 51–53]. Diarrhoea hospitalisation increased by 6.7% (95% CI: 4.6%– 8.9%) and 8.3% (95% CI: 3.1%– 13.1%) on a TAV95 and TAV99 heat wave day.

On the other hand, diarrhoea hospitalisation decreased by 3.5% and 4.9% three days following a TAV95 and TAV99 heat wave day, respectively. The apparent protective incidence rate ratio obtained at the lag of 3 days suggested some degree of short-term morbidity displacement i.e. ‘harvesting’ effect. During heat waves, excess hospitalisation due to recent heat wave day (lag 3) may be offset by deficits due to diarrhoea hospitalisation accelerated a couple of days by previous heat wave days. A study in Vietnam has reported short-term displacement effect of diarrhoeal diseases due to rainfall [54]. The present study was the first to detect any harvesting effect due to extreme heat on diarrhoea hospitalisation. A previous study investigating the effect of heat waves on infectious diarrhoea in Zurich reported a more pronounced effect of heat wave when a 7-day delayed effect of heat waves was considered. The same study reported an immediate effect of heat wave on diarrhoea due to inflammatory bowel disease (IBD). However, no harvesting effect of heat waves was identified in that study [46]. In contrast, this study identified an both immediate and harvesting effect of heat waves on diarrhoea hospitalisation in Bangladesh.

Heat waves can promote environmental expansion of diarrhoeal pathogens, increase consumption of contaminated drinking water and/or increase food spoilage leading to excess diarrhoea [8, 39, 40]. Given that a few previous studies have reported lags of days between dates of onset of diarrhoea and healthcare seeking in affected individuals, heat wave driven diarrhoea hospitalisation may be expected to take a few days to occur. However, the effects of extreme temperature on diarrhoea hospitalisation were mostly immediate in this study. One previous study using data from the same hospital in Dhaka reported that most of the severely dehydrated patients presented to the hospital within a narrow window of only 4–12 hours after symptom onset [55]. This suggested that the hospitalised patients in this study likely presented to the hospital on the same day of the symptom onset. This may partly explain the observed immediate effect of heat waves on diarrhoea hospitalisation in Dhaka. In addition, heat waves may aggravate infectious diarrhoea among already affected individuals leading to the excess hospitalisation for diarrhoea on the same day. While most of the patients enrolled in this study are likely to be infectious in origin, a few IBD diarrhoea cases may have been enrolled. Given that physical and mental stress can lead to flares of IBD and because heat stress are known to increase the frequencies of stress-dependent events including heart attacks and heat strokes [56], heat waves may trigger the flares of IBD or worsen a clinically non-apparent flare leading to excess diarrhoea [57].

In general, the effects of heat waves were most intense for children under 5 years of age compared to all ages. While the exact mechanism by which extreme temperature affect children’s vulnerability to diarrhoea has never been investigated in much detail, children may be generally more susceptible to infections owing to their immature immune systems and low self-care capacity [35, 58–60].

In this study, heat wave days defined by the exceedance of both 95^th^ and 99^th^ percentile of both daily mean temperature and daily maximum temperature performed as significant predictors of diarrhoea hospitalisation. A previous study investigating the effects of heat waves on mortality proposed the heat wave indicator combining day and night time temperatures as a suitable catchall indicator for heat waves in Bangladesh [9]. However, D&N heat wave day was only significantly associated with childhood diarrhoea in the present study. The findings of the present study therefore suggest that D&N heat wave may not serve as a suitable indicator for heat wave in Dhaka, Bangladesh in relation to diarrhoeal disease morbidity. Although high nighttime temperatures (i.e. daily minimum temperature) are known to precipitate heat-related mortality by providing no cooling-down period at night [10], such effects may not be relevant to diarrhoeal disease context and expectedly high minimum temperature was not found to be significantly associated with diarrhoea hospitalisation in this study.

While the robust surveillance system, clinical diagnoses, 30-year duration and the relative completeness of coverage of Dhaka’s population constitute key strengths of the data set used in this study, there are several limitations. The estimated total number of all-cause diarrhoea cases hospitalised per day may not represent the exact number of cases admitted in the icddr,b Dhaka Hospital. Furthermore, the less severe cases were less likely to be included. In addition, the study used estimated data from one hospital in one city given that reliable records were not available and/or accessible during the study period. However, these issues do not pose a threat to the validity of the analysis of trends and comparisons of heat wave-diarrhoea relationships over time, which is the theme of this study. Furthermore, there is no reason to expect the effects of heat wave to vary significantly in patients from Dhaka seeking care from hospitals other than icddr,b Dhaka Hospital. In addition, numerous models were evaluated in this study during the sensitivity analyses to check the robustness of the results. Although the robustness of the results to varying degrees of control for long-term trend and seasonality was reassuring, yet there remains some possibility of residual confounding. Furthermore, there are uncertainties related to the extrapolation of the relationships revealed in this study to other locations with different climate and geography. In particular, the observed association may also be greatly dependent on other important factors including the degree of water and sanitation infrastructure and hygiene practices in an area. Furthermore, heat wave effects on diarrhoea may vary by causative organisms [39, 40]. Future studies using pathogen-specific and recent data may generate better estimates and help to validate the findings from this study. Additionally, studies from different geographic locations and socio-economic settings may provide additional information if the findings would pertain to other places.

## Conclusion

This study identified heat wave as a risk factor for diarrhoea hospitalisation in Dhaka, Bangladesh by proposing several heat wave indices. TAV95 is the preferred heat wave indicator, which defines a heat wave as the elevated daily mean temperature above the 95^th^ percentile persisting for at least one day. This definition results in 409 heat wave days and 176 separate heat waves in 30 years from 1981 to 2010. Almost all the heat waves occurred during the pre-monsoon summer season, between April and June, with the highest number of heat waves in May. Diarrhoea hospitalisations increased by 7% in all ages and 14% among children under 5 years of age during a TAV95 heat wave day compared to a non-heat wave day. These results can be used to define heat waves for Dhaka and motivate public health interventions including generation of heat alerts to prevent heat-related morbidity in Dhaka.

## Supporting information

S1 Checklist Inclusivity in global research. (DOCX)

S1 File Additional information on health data. (DOCX)

S2 File Findings from the exploratory analyses. (DOCX)

S3 File Findings from the sensitivity analyses. (DOCX)
