# Supplementary material for: Is heat wave a predictor of diarrhoea in Dhaka, Bangladesh? A time-series analysis in a South Asian tropical monsoon climate
Source: PLOS Glob Public Health. 2024 Sep 3;4(9):e0003629. doi: 10.1371/journal.pgph.0003629 (PMC11371214; doi:10.1371/journal.pgph.0003629)
Supplement: S2 File — (DOCX) [file pgph.0003629.s003.docx]

**S2 File. Exploratory analyses.**

Exploratory analyses were conducted initially to understand the time series characteristics of each meteorological and health data series using established methods (1-3). Exploratory analyses began with the exploration of missing data. Specifically, the techniques for visualizing time series data such as time series plots were used to reveal high-level patterns in the data. Moving average plots that effectively smooth out the raw data by averaging over a fixed number of adjacent raw data points were also generated. Summary statistics were estimated, the correlation matrix was generated and missing data was explored (3).

Time series data sets frequently show non-stationarity and auto-correlation. Non-stationarity means that the data cannot maintain a constant mean and variance across different time frames so that the expected count and variation about it change over time, whereas autocorrelation is defined as the dependence between successive observations in a time series. Time series data also frequently reveal long-term patterns, seasonality, overdispersion and heteroscedasticity. Overdispersion occurs when the variance of response is greater than the nominal variance in discreet-response models. On the other hand, heteroscedasticity means that the residuals belong to a population that does not have a constant variance. In addition, time series data frequently lack normality. Problems of non-stationarity, autocorrelation, heteroscedasticity, lack of normality and overdispersion in the data violate several assumptions of traditional linear regression models. As a result, each univariate time series data need to be checked for stationarity, autocorrelation, long-term trends, seasonality, overdispersion, possible outliers, heteroscedasticity and normality before building any models (3, 4).

In this study, visual plots were used as an initial tool to identify trend, outliers, seasonality, long-run cycle, constant variance (i.e. stationarity) and any abrupt changes (volatility) in each data series. The Augmented Dickey Fuller test that can check the correlation in error terms by adding lags was then utilised to formally determine any non-stationarity in the data. As a preliminary step, the first or second difference of the series was attempted to resolve the issue of non-stationarity when identified visually in any data series. Visual plots of the differenced data and Augmented Dickey Fuller test was performed again on the differenced data to check for stationarity before proceeding forward with further time series analyses (3).

Similarly, autocorrelation was initially diagnosed visually using correlogram (sample autocorrelation function ACF plots) and tested using the Durbin Watson D test and Breusch Godfrey LM test. The prais and corc options were utilised to initially correct autocorrelation. The prais uses the generalized least-squares method to estimate the parameters in a linear regression model in which the errors are serially correlated. Specifically, the errors are assumed to follow a first-order autoregressive process. The corc specifies that the Cochrane–Orcutt transformation be used to estimate the equation. With this option, the Prais–Winsten transformation of the first observation is not performed, and the first observation is dropped when estimating the transformed equation.

As a preliminary tool, the lag order selection statistic in Vector Auto Regression (VAR) was used to identify the possible lag structure between the dependent and the independent variables. Johansen co-integration test was also performed to determine if the two time series were co-integrated (3). The sample cross correlation function (CCF) was ultimately used for identifying lags of the *x*-variable that might be useful predictors of *Y_t._* Since the CCF pattern is affected by the underlying time series structures of the dependent and independent variables, prewhitening was performed to resolve this issue. Prewhitening is an operation that processes a time series to make it behave statistically like white noise. Prewhitening is the process of eliminating or reducing short-term stochastic persistence to enable detection of deterministic change that has been extensively applied to time series analysis. Each data series was pre-whitened to achieve stationarity before checking the cross-correlation between the dependent variable (for example, daily hospitalisations for diarrhoeal diseases) and the predictors (such as daily ambient temperature). The univariate autoregressive integrated moving average (ARIMA) structure of the independent variable was taken into account before graphing the CCF. All possible AR, MA and I terms were identified using the combined ACF and partial autocorrelation function (PACF) plots. All possible ARIMA models were developed. The ARIMA model with the lowest information criteria (BIC and/or AIC) values was chosen as the best possible ARIMA model of the independent variable and the residuals from that model was stored. The dependent variable series was then filtered using the stored independent variable model. Then CCF between the residuals of the filtered dependent and independent variables were generated to identify the possible terms for a lagged regression. Prewhitening was only used to aid identification of which lags of the independent variables predicted the dependent variable. After identifying the possible model from the CCF, the original variables were used to estimate the lagged regression (3, 5). Given that there is no consensus on the best approach for choosing the lags, alternative strategies to prewhitening that includes looking at the CCF of the original variables and detrending the series using either the first differences or linear regressions with time as a predictor were also explored. Evidence from past studies and biological plausibility were also taken into account while choosing appropriate lags (3).

**Findings**

There were an estimated 2,983,850 hospital admissions for diarrhoeal disease in the 10,957 days from 1 January 1981 to 31 December 2010. Among these, 58% were males. 54% were aged <5 years, 10% were aged 5−14 years and 36% were ≥15 years. S1 Table shows the summary of the missing data, which reveals that the majority of the days of observations were available with very little missing data for all variables.

**S1 Table Summary of missing data**

| Time series variable (Unit) | Total number of days of observations | Total number of days with missing data (proportions) |
| --- | --- | --- |
| Daily temperature (°C) | | |
| Ambient | 10,957 | 0 (0.0000) |
| Maximum | 10,928 | 29 (0.0026) |
| Minimum | 10,946 | 11 (0.0010) |
| Daily relative humidity (%) | 10,954 | 3 (0.0003) |
| Daily cumulative rainfall (mm) | 10,954 | 3 (0.0003) |
| Daily diarrhoea admissions | | |
| All ages all-cause | 10,925 | 32 (0.0029) |
| <5 years of age | 10,925 | 32 (0.0029) |

The summary statistics for the mean, maximum, minimum temperatures, relative humidity, and total and age-group specific diarrhoea admissions are displayed in the S2 Table. All temperature measures showed slight increase over the decades. Daily diarrhoea hospitalisations almost doubled during 1991-2010 compared to 1981–1990.

S1 Figure shows the temporal distribution of all-cause diarrhoea cases in all ages during the study period. The time series plot of total daily hospital visits for all-cause diarrhoea in the icddr,b Dhaka Hospital, showed that there was possibly an upward trend and seasonality. However, the graph is only a preliminary step in formal test process of stationarity. Whether the variance was constant or non-constant and whether there was any long-term cycle could not be ascertained. There appeared to be no abrupt changes or any major outliers. The monthly time series plot suggested a strong seasonal pattern with dual peaks in hospitalisations during the months of April and September.

S2 S3 Figures show the temporal distribution of daily mean, minimum, maximum and dew point temperature that display strong seasonal patterns. Similarly, daily relative humidity and cumulative rainfall in S2 Figure reveal a seasonal pattern.

**S2 Table Distribution of the daily meteorological parameters in Dhaka, and the number of hospital admissions in icddr,b Dhaka Hospital between 1 January 1981 and 31 December 2010**

| Period | **1981 - 1990** | | **1991 - 2000** | | **2001 - 2010** | | **1981 - 2010** | |
| --- | --- | --- | --- | --- | --- | --- | --- | --- |
| Variable (unit) | 25^th^ -75^th^ percentile | Median | 25^th^ -75^th^ percentile | Median | 25^th^ -75^th^ percentile | Median | 25^th^ -75^th^ percentile | Median |
| Daily temperature (^o^C) |  |  |  |  |  |  |  |  |
| Ambient | 22.8 - 29.0 | 27.1 | 22.7 - 29.1 | 27.2 | 23.0 - 29.2 | 27.5 | 22.8 - 29.1 | 27.3 |
| Maximum | 28.6 - 33.0 | 31.2 | 28.5 - 33.2 | 31.5 | 28.5 - 33.4 | 31.5 | 28.5 - 33.2 | 31.4 |
| Minimum | 17.4 - 26.1 | 23.4 | 17.4 - 26.0 | 23.6 | 18.2 - 26.0 | 23.7 | 17.6 - 26.1 | 23.6 |
| Daily relative humidity (%) | 70 - 83 | 77 | 71 - 82 | 77 | 67 - 81 | 74 | 69 - 82 | 76 |
| Daily cumulative rainfall (mm) | 0 - 3 | 0 | 0 - 3 | 0 | 0 - 2 | 0 | 0 - 3 | 0 |
| Daily diarrhoea admissions |  |  |  |  |  |  |  |  |
| All ages | 150 - 225 | 175 | 250 - 375 | 300 | 200 - 350 | 300 | 200 - 325 | 250 |
| <5 years of age | 75 - 125 | 100 | 125 - 200 | 150 | 100 - 200 | 150 | 100 - 200 | 150 |

**S1 Figure Daily, monthly and yearly distribution (raw plots) of hospital admissions for all-cause diarrhoea in all ages at the icddr,b Dhaka Hospital between 1 January 1981 and 31 December 2010.**

**S2 Figure Daily distribution of mean (upper left), minimum (upper right), maximum (middle left), relative humidity (middle left) and cumulative rainfall (lower left) in Dhaka, Bangladesh 1 January 1981 and 31 December 2010.**

**S3 Figure Monthly distributions of mean, maximum, minimum and dew point temperatures in Dhaka, Bangladesh between 1 January 1981 and 31 December 2010**


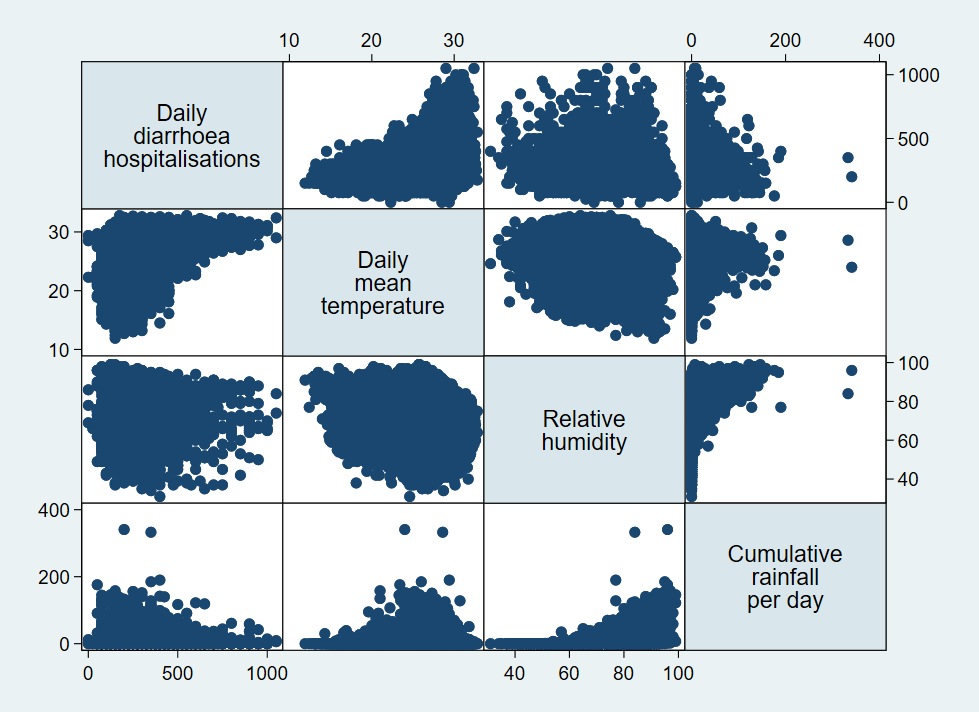


**S4 Figure Correlation matrix showing the crude relationship between daily diarrhoea hospitalisations with different meteorological parameters**

S4 Figure shows that all-cause diarrhoea hospitalisation, daily mean temperature, relative humidity and cumulative rainfall are correlated.

**S3 Table Augmented Dickey-Fuller test for unit root with trend term in regression**

| Time series variable | Test statistic Z(t) | Dickey-Fuller critical value | | | Mackinnon approximate *p*-value for z(t) |
| --- | --- | --- | --- | --- | --- |
|  |  | 1% | 5% | 10% |  |
| All-cause diarrhoea hospitalisation | -27.446 | -3.960 | -3.410 | -3.120 | 0.0001 |
| First differenced all-cause diarrhoea | -169.568 | -3.430 | -2.860 | -2.570 | 0.0000 |
| Second differenced all-cause diarrhoea | -229.908 | -3.430 | -2.860 | -2.570 | 0.0000 |
| Under 5 diarrhoea hospitalisation | -67.979 | -3.960 | -3.410 | -3.120 | 0.0000 |
| Daily mean temperature | -16.281 | -3.960 | -3.410 | -3.120 | 0.0000 |
| Daily maximum Temperature | -27.621 | -3.960 | -3.410 | -3.120 | 0.0000 |
| Daily minimum temperature | -18.154 | -3.960 | -3.410 | -3.120 | 0.0000 |
| Daily cumulative rainfall | -76.662 | -3.960 | -3.410 | -3.120 | 0.0000 |
| Daily relative humidity | -32.583 | -3.960 | -3.410 | -3.120 | 0.0000 |

The Augmented Dickey Fuller test, which checks the correlation in error term by adding lags, was conducted to formally determine the stationarity of each series. The stationarity of each series was examined by focusing on only two values of the result; Z(t) and Mackinnon p-value for Z(t). Given that the Z(t) values for all variables have large negative numbers and the p-values are significant, the null hypothesis of Dickey Fuller test, which states that the time series data is non-stationary, can be rejected. As revealed in S3 Table, all the time series data for all-cause diarrhoea in all ages and in children under 5 years of age as well as the meteorological parameters are stationary even after taking lags for correlated error terms.

S5 Figure shows the smoothed relationship between all-cause diarrhoea hospitalisation in all ages with daily mean temperature suggesting a linear positive relationship. The figure also suggests linear negative relationship between diarrhoea hospitalisation and daily relative humidity. The smoothed relationship between daily diarrhoea hospitalisation and cumulative rainfall is unclear.

S6 and S7 Figures show the autocorrelation and partial autocorrelation functions of the daily diarrhoea hospitalisation series. The PACF plot (S6 and S7 Figures) reveal a tapering pattern that becomes non-significant after lags of 7 days. However, lags 1, 2 and 3 have much larger values compared to the other significant lags. The ACF plot shown in S6 and S7 figures exhibit large persistent sample autocorrelation functions that decay linearly. This suggest that the series is not integrated and hence an ARMA process could be used to pre-whiten the daily diarrhoea hospitalisation data series. The combined ACF and PACF were used to identify possible ARIMA (p,q,d) models. Accordingly, the data series was pre-whitened using various ARMA models as highlighted in S4 Table. Among the various models, the ARIMA model (2,0,2) displayed the lowest BIC value. The ACF and PACF of the residuals of the model had no significant values at any lags suggesting that the model would be appropriate for prewhitening the all-ages diarrhoea data series.

**S5 Figure Smoothed relationship of all-cause diarrhoea hospitalisations with daily mean temperature, relative humidity and cumulative rainfall**

**S6 Figure Correlogram of daily diarrhoea hospitalisation**

**S7 Figure Plots of autocorrelation function and partial autocorrelation function of daily all-cause diarrhoea hospitalisations in all ages**

**S4 Table Comparison of ARMA models for time series of daily diarrhoea hospitalisation**

|  | (1,0,1) | (1,0,2) | (1,0,3) | (2,0,1) | (2,0,2) | (2,0,3) | (3,0,1) | (3,0,2) |
| --- | --- | --- | --- | --- | --- | --- | --- | --- |
| Cons | 272.30 | 272.30 | 272.30 | 272.29 | 272.33 | 272.33 | 272.30 | 272.30 |
| L1.ar | 0.985 | 0.985 | 0.984 | 0.947 | 1.882 | 1.874 | 0.919 | 0.332 |
| L2.ar |  |  |  | 0.037 | -0.884 | -0.876 | 0.039 | 0.603 |
| L3.ar |  |  |  |  |  |  | 0.025 | 0.039 |
| L1.ma | -0.586 | -0.599 | -0.599 | -0.561 | -1.501 | -1.491 | -0.533 | 0.054 |
| L2.ma |  | 0.023 | 0.013 |  | 0.556 | 0.543 |  | -0.334 |
| L3.ma |  |  | 0.016 |  |  | 0.006 |  |  |
| AIC | 115598 | 115594 | 115593 | 115594 | 115566* | 115567 | 115594 | 115597 |
| BIC | 115627 | 115630 | 115637 | 115631 | 115609* | 115618 | 115638 | 115648 |

**References**

1. Bhaskaran K, Gasparrini A, Hajat S, Smeeth L, Armstrong B. Time series regression studies in environmental epidemiology. Int J Epidemiol. 2013;42(4):1187-95.

2. Imai C, Armstrong B, Chalabi Z, Mangtani P, Hashizume M. Time series regression model for infectious disease and weather. Environ Res. 2015;142:319-27.

3. Shumway RH, Stoffer DS, editors. Time Series Analysis and Its Applications Fourth ed. Switzerland Springer International Publishing AG; 2017.

4. Hardin JW, Hilbe JM, editors. Generalized Linear Models and Extensions 2nd ed. Texas 77845: Stata Press; 2007.

5. Becketti S, editor. Introduction to Time Series Using Stata. Revised ed. College Station, Texas Stata Press 2020.
