# Supplementary material for: Is heat wave a predictor of diarrhoea in Dhaka, Bangladesh? A time-series analysis in a South Asian tropical monsoon climate
Source: PLOS Glob Public Health. 2024 Sep 3;4(9):e0003629. doi: 10.1371/journal.pgph.0003629 (PMC11371214; doi:10.1371/journal.pgph.0003629)
Supplement: S3 File — (DOCX) [file pgph.0003629.s004.docx]

**S3 File. Sensitivity analyses.**

Sensitivity analyses were conducted by varying the amount of control for long-term trend and seasonality in model with TAV95 heat wave day indicator. Initially, the analyses were repeated using linear splines (LS) of 1-12 degrees of freedom per year to control for long-term trend and seasonality to check the robustness of the results.

For the sensitivity analysis, the model took the following form:

*Y_t_ ~ Negative Binomial (µt, θ)*

log[E(*Y_t_*)]= β_0_+ ∑β*_1p_ET_t-1p_* + ∑β*_2q_HeavyRain_t-2q_* + ∑LS(*time_t ,_* 1-12 D.F.)+ ∑ β*_3_DOW* ……………………………………………..……………….(1-1)

To allow for seasonality and long-terms trends in the data, natural cubic spline (NS) function of time was fitted instead of linear spline. The analyses were repeated using 3–7 degrees of freedom per year for calculating the number of knots to estimate whether the results were sensitive to the levels of control for the long-term trend and seasonal patterns.

For the sensitivity analysis, the model took the following form:

*Y_t_ ~ Negative Binomial (µt, θ)*

log[E(*Y_t_*)]= β_0_+ ∑β*_1p_ET_t-1p_* + ∑β*_2q_HeavyRain_t-2q_* + ∑NS(*time_t ,_* 3-7 D.F.)+ ∑ β*_3_DOW* ………………….………………………………….....……..(1-2)

where Y_t_ denoted daily all-cause diarrhoea count, ET_t_ and HeavyRain_t_ denoted heat extreme and heavy rainfall indicator at time t. To control for long-term trends and seasonality, a natural cubic spline (NS) of time with 7 degrees of freedom per year was incorporated into the model. DOW_t_ was the categorical day of the week with a reference day of Friday.

Further analysis was conducted by including relative humidity as a linear term and heavy rainfall as a categorical variable without any lagged effects.

For the sensitivity analysis, the model took the following form:

*Y_t_ ~ Negative Binomial (µt, θ)*

log[E(Yt)]= β_0_ + β_1_ET_t_ + β_2_HeavyRain_t_ + β_3_Hum_t_ + ∑ β_4_DOW_t_  + ∑ NS(*time_t_*, 7 D.F.)…………………………………………………………….(1-3)

To explore to what extent the changes in the sample size affected the results, the models were re-run using the actual number of diarrheal disease patients enrolled into the surveillance system (DDSS) instead of the total estimated number of patients with diarrheal diseases in the Dhaka Hospital during the study period.

**log[E(*DDSS_t_*)]= β_0_+ ∑β*_1p_ET_t-1p_* + ∑β*_2q_HeavyRain_t-2q_* + ∑NS(*time_t_* *_,_* 7 D.F.)+ ∑ β*_3_DOW* ………………………………….………………..……….(1‑4)**

**Findings**

The results of the sensitivity analysis are shown in 1S Table. All results are comparable to the primary analysis (2S Table). The models with the lowest BIC values were considered as the best models.

**1S Table. IRR of diarrhea hospitalisation in all ages and <5 children during a TAV95 heat wave day in Dhaka Bangladesh**

|  | **Incidence Rate Ratio IRR (95% CI)** | | | | | |
| --- | --- | --- | --- | --- | --- | --- |
|  | **All ages** | | | **Under-5 children** | | |
| D.F. | Using Equation 1-1 | BIC | Dispersion |  | BIC | Dispersion |
| 1 | 1.0459 (1.0254 – 1.0668) | 7601.0 | 1.10 | 1.1191 (1.0681 – 1.1973) | 7261.5 | 1.11 |
| 2 | 1.0415 (1.0217 – 1.0617) | 7601.1 | 1.07 | 1.1251 (1.0711 – 1.1903) | 7264.0 | 1.10 |
| 3 | 1.0445 (1.0242 – 1.0652) | 7600.9 | 1.07 | 1.1291 (1.0781 – 1.2073) | 7258.2 | 1.10 |
| 4 | 1.0411 (1.0210 – 1.0616) | 7601.0 | 1.08 | 1.1321 (1.0756 – 1.2070) | 7254.9 | 1.10 |
| 5 | 1.0496 (1.0293 – 1.0703) | 7600.5 | 1.05 | 1.1351 (1.0712 – 1.1793) | 7255.3 | 1.08 |
| 6 | 1.0485 (1.0284 – 1.0691) | 7600.5 | 1.06 | 1.1372 (1.0751 – 1.2004) | 7255.2 | 1.04 |
| 7 | 1.0486 (1.0285 – 1.0691) | 7600.5 | 1.06 | 1.1381 (1.0771 – 1.2051) | 7230.1 | 1.04 |
| 8 | 1.0509 (1.0311 – 1.0711) | 7597.1 | 0.96 | 1.1391 (1.0781 – 1.2073) | 7221.2 | 1.02 |
| 9 | 1.0500 (1.0302 – 1.0701) | 7601.1 | 0.92 | 1.1371 (1.0756 – 1.2023) | 7235.5 | 1.09 |
| 10 | 1.0585 (1.0384 – 1.0791) | 7603.1 | 0.89 | 1.1322 (1.0701 – 1.2017) | 7258.9 | 1.12 |
| 11 | 1.0631 (1.0331 – 1.0734) | 7609.8 | 0.81 | 1.1341 (1.0722 – 1.2093) | 7257.8 | 1.09 |
| 12 | 1.0670 (1.0371 – 1.0773) | 7609.9 | 0.75 | 1.1297 (1.0781 – 1.2052) | 7300.9 | 1.07 |
| D.F. | Using Equation 1-2 |  |  |  |  |  |
| 3 | 1.0690 (1.0081 – 1.0803) | 7600.2 | 0.82 | 1.1314 (1.0738 – 1.2132) | 7200.4 | 1.09 |
| 4 | 1.0608 (1.0098 – 1.0821) | 7600.2 | 0.85 | 1.1321 (1.0744 – 1.2041) | 7185.7 | 1.10 |
| 5 | 1.0690 (1.0182 – 1.0801) | 7599.8 | 0.83 | 1.1332 (1.0798 – 1.1955) | 7199.8 | 1.07 |
| 6 | 1.0624 (1.0207 – 1.0837) | 7598.0 | 0.81 | 1.1344 (1.0828 – 1.2063) | 7198.9 | 1.06 |
| 7* | 1.0672 (1.0460 – 1.0889) | 7596.4 | 0.96 | 1.1393 (1.0827 – 1.1988) | 7125.8 | 1.01 |
| D.F | Using Equation 1-3 |  |  |  |  |  |
| 7 | 1.0530 (1.0220 – 1.0730) | 7599.9 | 0.98 | 1.1299 (1.0854 – 1.1996) | 7245.8 | 1.05 |
| D.F. | Using Equation 1-4 |  |  |  |  |  |
| 7 | 1.0622 (1.0254 – 1.0891) | 7599.7 | 0.97 | 1.1354 (1.0838 – 1.1997) | 7245.6 | 1.05 |

*Represents the models with the lowest BIC values.

NB: Dispersion value near unity represent better fit; dispersion<1 represent under-parameterization; dispersion>1 represent over-parameterization.

**2S Table. Percentage increase in diarrhoea hospitalisation in all ages and <5 children during heat wave days compared to non-heat wave days in Dhaka, 1981–2010 (with model evaluation statistics included)**

| Indicator | All ages | | | | <5 Children | | | |
| --- | --- | --- | --- | --- | --- | --- | --- | --- |
|  | Percentage increase in diarrhoea hospitalisations on heat wave days (95% CI) | P-value | BIC | Dispersion | Percentage increase in diarrhoea hospitalisations on heat wave days (95% CI) | P-value | BIC | Dispersion |
| TAV95 | 6.7 (4.6 – 8.9) | <0.001 | 7596.4 | 0.9577 | 13.9 (8.3 – 19.9) | <0.001 | 7949.0 | 1.0167 |
| TAV99 | 8.3 (3.7 – 13.1) | <0.001 | 7597.8 | 0.9567 | 24.2 (11.3 – 38.7) | <0.001 | 7950.4 | 1.0167 |
| D95 | 7.0 (4.8 – 9.3) | <0.001 | 7597.2 | 0.9588 | 17.0 (11.0 – 23.5) | <0.001 | 7947.3 | 1.0171 |
| D99 | 7.4 (3.1 – 11.9) | 0.001 | 7599.1 | 0.9600 | 19.5 (7.7 – 32.6) | 0.001 | 7950.1 | 1.0167 |
| MIN95 | 0.05 (-0.2 – 2.1) | 0.964 | 7599.5 | 0.9606 | 4.4 (-0.8 – 9.9) | 0.098 | 7951.6 | 1.0164 |
| D&N | 4.0 (-0.8 – 9.1) | 0.107 | 7598.8 | 0.9577 | 14.0 (0.9 – 28.7) | 0.035 | 7951.5 | 1.0164 |
| TAV952 | 4.6 (0.4 – 9.0) | 0.031 | 7598.0 | 0.9571 | 21.0 (3.2 – 41.9) | 0.019 | 7951.2 | 1.0165 |
| TAV953 | -1.3 (-9.3 – 7.5) | 0.770 | 7799.4 | 0.9580 | 17.0 (-5.7 – 45.2) | 0.153 | 7951.8 | 1.0164 |
| TAV992 | 1.9 (-0.50 – 9.2) | 0.599 | 7799.9 | 0.9581 | 29.4 (-3.9 – 74.3) | 0.089 | 7951.5 | 1.0165 |
| TAV993 | 5.2 (-3.8 – 15.1) | 0.269 | 7799.6 | 0.9577 | 16.7 (-27.0 – 86.5) | 0.519 | 7952.0 | 1.0164 |
| D952 | 1.9 (-5.0 – 9.2) | 0.599 | 7599.9 | 0.9581 | 13.4 (-4.91 – 35.1) | 0.162 | 7951.7 | 1.0160 |
| D953 | 5.2 (-3.8 – 15.1) | 0.269 | 7599.6 | 0.9577 | 22.3 (-2.7 – 53.6) | 0.084 | 7951.5 | 1.0165 |
| D992 | 11.5 (-2.3 – 27.2) | 0.755 | 7599.7 | 0.9585 | 22.77 (-12.1 – 71.3 | 0.230 | 7951.7 | 1.0164 |
| D993 | -34.1 (-5.2 – 36.5) | 0.776 | 7599.9 | 0.9578 | -11.8 (-65.4 – 25.2) | 0.793 | 7952.0 | 1.0164 |
| MIN952 | -5.8 (-11.6 – 0.2) | 0.058 | 7599.8 | 0.9581 | -2.3 (-16.5 – 14.4) | 0.755 | 7952.0 | 1.0164 |
| D&N2 | -4.8 (-19.4 – 12.5) | 0.562 | 7599.3 | 0.9573 | 19.4 (-21.6 – 81.9) | 0.409 | 7951.7 | 1.0164 |

**References**

1. Hardin, J.W. and J.M. Hilbe, eds. *Generalized Linear Models and Extensions* 2nd ed. 2007, Stata Press: Texas 77845.

2. Hardin JW, Hilbe JM, editors. Generalized Linear Models and Extensions. Fourth ed. Texas: StataCorp LP; 2018.

3. Chandler RE, Scottt EM, editors. Statistical Methods for Trend Detection and Analysis in the Environmental Sciences. First ed. United Kingdom John Wiley & Sons, Ltd; 2011.

4. Imai C, Armstrong B, Chalabi Z, Mangtani P, Hashizume M. Time series regression model for infectious disease and weather. Environ Res. 2015;142:319-27.

5. Becketti S, editor. Introduction to Time Series Using Stata. Revised ed. College Station, Texas Stata Press 2020.

6. Bhaskaran K, Gasparrini A, Hajat S, Smeeth L, Armstrong B. Time series regression studies in environmental epidemiology. Int J Epidemiol. 2013;42(4):1187-95.
